# Enhancing the capacity of the mental health and substance use health workforce to meet population needs: insights from a facilitated virtual policy dialogue

**DOI:** 10.1186/s12961-022-00857-8

**Published:** 2022-05-07

**Authors:** Kathleen Leslie, Mary Bartram, Jelena Atanackovic, Caroline Chamberland-Rowe, Christine Tulk, Ivy Lynn Bourgeault

**Affiliations:** 1 Faculty of Health Disciplines, Athabasca University, 1 University Drive Athabasca, Athabasca, AB T9S 3A3 Canada; 2 Mental Health Commission of Canada, 350 Albert Street, Suite 1210, Ottawa, ON K1R 1A4 Canada; 3 School of Sociological and Anthropological Studies, University of Ottawa, 75 Laurier Avenue East, Ottawa, ON K1N 6N5 Canada; 4 Telfer School of Management, University of Ottawa, 75 Laurier Avenue East, Ottawa, ON K1N 6N5 Canada; 5 Faculty of Arts and Social Sciences, Carleton University, 1125 Colonel By Drive, Ottawa, ON K1S 5B6 Canada

**Keywords:** Virtual policy dialogue, Knowledge mobilization, Stakeholder participation in research, Evidence-informed policy, COVID-19

## Abstract

**Background:**

Timely knowledge mobilization has become increasingly critical during the COVID-19 pandemic and complicated by the need to establish or maintain lines of communication between researchers and decision-makers virtually. Our recent pan-Canadian research study on the mental health and substance use health (MHSUH) workforce during the pandemic identified key policy barriers impacting this essential workforce. To bridge the evidence–policy gap in addressing these barriers, we held a facilitated virtual policy dialogue. This paper discusses the insights generated at this virtual policy dialogue and highlights how this integrated knowledge mobilization strategy can help drive evidence-based policy in an increasingly digital world.

**Methods:**

We held a 3-hour virtual policy dialogue with 46 stakeholders and policy decision-makers as the final phase in our year-long mixed-methods research study. The event was part of our integrated knowledge mobilization strategy and was designed to generate stakeholder-driven policy implications and priority actions based on our research findings. The data collected from the virtual policy dialogue included transcripts from the small-group breakout rooms and main sessions, reflective field notes and the final report from the external facilitator. Coded data were thematically analysed to inform our understanding of the prioritization of the policy implications and action items.

**Results:**

Facilitated virtual policy dialogues generate rich qualitative insights that guide community-informed knowledge mobilization strategies and promote evidence-informed policy. Our policy dialogue identified actionable policy recommendations with equity as a cross-cutting theme. Adapting policy dialogues to virtual formats and including technology-assisted facilitation can offer advantages for equitable stakeholder participation, allow for deeper analysis and help build consensus regarding evidence-based policy priorities.

**Conclusions:**

Our facilitated virtual policy dialogue was a key knowledge mobilization strategy for our research on the capacity of the Canadian MHSUH workforce to respond to the COVID-19 pandemic. Our policy dialogue allowed us to engage a diverse group of MHSUH workforce stakeholders in a meaningful action-oriented way, provided an avenue to get feedback on our research findings, and generated prioritized action items that incorporated the knowledge and experience of these MHSUH workforce stakeholders.

**Supplementary Information:**

The online version contains supplementary material available at 10.1186/s12961-022-00857-8.

## Background

Timely knowledge mobilization is a growing concern in social science and health research. This has become increasingly critical during the pandemic and complicated by the need to establish or maintain lines of communication between researchers and decision-makers virtually. Our study on the mental health and substance use health (MHSUH) workforce was funded by a Canadian Institutes of Health Research COVID-19 grant and illustrates promising practices in timely knowledge mobilization using rich qualitative data from a facilitated virtual policy dialogue.

### The capacity of the MHSUH workforce

The MHSUH workforce is in critical demand. MHSUH needs and concerns have become even more widespread across Canada during the COVID-19 pandemic and may continue to increase through the anticipated echo pandemic, as the lingering impacts of financial stress, social isolation and bereavement take their toll [1, 2]. While 67% of Canadians reported excellent or very good mental health in 2019, this percentage has dropped to 40% during the pandemic [3]. Similarly, the prevalence of depression symptoms has significantly increased across the population (from 2 to 14%) [3]. Substance use has also increased, with one in four Canadians aged 35–54 years and one in five Canadians aged 18–34 years reporting increased alcohol consumption since the pandemic began [4]. Further, these MHSUH impacts have disproportionately affected groups with higher risk factors, including women with younger children, people who live alone, people with a previous diagnosis of a mental health or substance use disorder, youth, people who identify as 2SLGBTQ+, and people who have a low income or are unemployed [3–5]. The pandemic has also exacerbated the “already problematic gaps in culturally and linguistically appropriate care” ([6] p. 969) and disproportionately affected those already facing inequitable access to care [7–9].

Exacerbating rising population health needs, health system disruptions during the pandemic have impacted MHSUH service provision [6, 9–11]. One survey revealed that the COVID-19 pandemic disrupted mental health services in 93% of countries worldwide, while the demand for services is increasing [12]. Some health system responses, such as converting psychiatric inpatient units to COVID-19 units and redeploying MHSUH staff to work in other healthcare areas, have negatively impacted access to care [2, 13, 14]. The pandemic impacts have also been felt in the substance use sector, where many services have become discontinued or of limited availability/capacity [7, 15]. One of the key inputs of health system responsiveness to growing MHSUH needs is the availability and accessibility of qualified MHSUH providers. Unfortunately, in the Canadian context, where most MHSUH providers are either privately funded or only partially publicly funded, this sector of the health workforce is generally overlooked in health policy and research [16, 17].

There is a dearth of data on the MHSUH workforce in Canada relative to comparable countries such as the United States [18]. We secured funding from a rapid, targeted mental health COVID grant to begin to address this gap. Our 12-month mixed-methods study included a literature review, pan-Canadian survey of MHSUH providers, and key informant interviews. Our study provides a foundational snapshot of this otherwise hidden workforce, including its capacity to respond to emerging individual, community and population health needs in Canada. Our results highlight the complex, policy-relevant barriers to increasing the MHSUH workforce’s capacity to provide services, including differences across occupations, genders and funding models. In order to bridge the evidence–policy gap in addressing the needs of this workforce, we held a facilitated virtual policy dialogue as the culminating knowledge mobilization strategy for our research findings.

### Policy dialogues as a knowledge mobilization strategy

Policy dialogues constitute an “interactive knowledge-sharing mechanism” and allow research evidence to be brought together with the knowledge and experience of those who will be involved in, or affected by, policy-making on specific high-priority issues ([19] p. 2). Given our focus on generating actionable policy recommendations, we use the term *policy dialogues* throughout this paper; however, these processes are also described as deliberative dialogues or stakeholder dialogues. Policy dialogues are versatile strategies to elicit broad input from diverse stakeholders on policy-relevant research evidence and generate targeted policy directions [20]. As a knowledge mobilization strategy, such dialogues can be a powerful tool to address complex policy challenges such as those related to mental health policy, allowing for the consideration of empirical evidence alongside the knowledge and experience of stakeholder participants [21, 22]. As such, policy dialogues are a valuable strategy to help bridge the gap between research evidence and policy. There is, however, limited literature available on policy dialogues facilitated virtually. This paper, with its practical description of a virtual policy dialogue, highlights their benefits and drawbacks and offers insights on leading practices for this valuable knowledge mobilization strategy in an increasingly digital world.

## Methods

### Virtual policy dialogue design

We held a 3-hour virtual policy dialogue via Zoom in June 2021 as the final phase in our mixed-methods study on the capacity of the MHSUH workforce in Canada. The event was part of our integrated knowledge mobilization strategy and was designed to generate stakeholder-driven policy implications and priority actions based on our research findings. The objectives of the policy dialogue were to share the key findings of our research, assess and foster consensus regarding the policy implications of these findings, and identify priority action items to move towards evidence-informed policy-making. The format and plan for the policy dialogue were designed by the research team, the study’s pan-Canadian expert advisory committee, and an expert facilitator external to the research team, who was hired for the event (see Table 1 for policy dialogue agenda). We incorporated elements of nominal group technique, using web-based group decision support system (GDSS) technology that enabled the capture, ranking and prioritization of input from a group of participants to generate ideas and understand prioritization. Classic nominal group technique involves silent idea generation regarding a particular question, round-robin sharing of ideas, group discussion and ranking of ideas by individual vote [23, 24]. The nominal group method differs from the Delphi method, another well-established consensus-building strategy, in its structured, small-group, face-to-face interaction; whereas the Delphi is premised on anonymous and asynchronous input and feedback [25].

**Table 1:** Agenda for virtual policy dialogue

Time	Activities
10 minutes	Introductions, review agenda, discuss process, conduct consent
20 minutes	Discuss preliminary research findings from the mixed-methods study on MHSUH provider capacity during the pandemic
Focus 1: What are the policy implications of the research findings?	
45 minutes	Small-group breakout room: brainstorming and discussion
25 minutes	Full-group discussion: synthesizing and prioritizing the policy implications
10 minutes	Break
Focus 2: What are the key action items, recommendations and next steps?	
30 minutes	Small-group breakout room: brainstorming and discussion
35 minutes	Full-group discussion: synthesizing and prioritizing the key action items, recommendations and next steps
5 minutes	Wrap-up and thank attendees

Two weeks before our virtual policy dialogue, in preparation for the event, all participants received an evidence brief of our preliminary study findings on the impact of the pandemic on the capacity of the MHSUH workforce in Canada. Following a research presentation, participants were divided into virtual breakout rooms with six or seven participants in each room. Breakout rooms were preassigned to ensure a mix of stakeholder sectors in each group and to allow participants to speak in their preferred language (French or English). In each breakout room, discussion was facilitated by a team member who was familiar with the research study and trained in the use of the GDSS software. The small groups were asked to reflect on and discuss the possible policy implications of our research findings (*Focus 1*). Ideas were brainstormed using the online tool and participants were able to see the ideas generated on the screen. Each small group was then asked to collate their ideas as best as possible and identify their top three policy implications.

Following the small-group discussion, the external facilitator engaged the whole group in a discussion of the top three policy implications of each group, highlighting the key points, clarifying the possible policy directions and creating a synthesized list of the ideas generated. These ideas were then individually ranked by participants using the GDSS software to determine which policy directions were considered the highest priority. The same process was followed to identify and prioritize key action items and next steps based on the identified policy implications (*Focus 2*).

With some adaptation, our virtual policy dialogue retained most features of established in-person policy dialogue practices. According to Boyko et al. [27], deliberative stakeholder dialogues have three defining features: a constructive meeting environment, a purposeful mix of participants and the appropriate use of research evidence. Addressing a high-priority issue, pre-circulating a policy brief, skilled facilitation, not attributing comments to individuals (Chatham House Rule), not emphasizing a need for consensus, and follow-up activities to support actions are also features contributing to the success of policy dialogues [19, 28]. These and other elements were synthesized by Damani et al. [20] in their article on an in-person policy roundtable. Drawing on their work, we have compared our facilitated virtual policy dialogue to these key features and guiding principles in Table 2. The one significant difference was that our dialogue explicitly built toward consensus. In a virtual environment, with less time and space for generative discussion, a technology-assisted and facilitated priority-setting activity helped to drive the discussion toward a more concrete outcome.

**Table 2:** Design elements of our facilitated virtual policy dialogue (adapted from Damani et al. [20])

Design element	Present?	Details and adaptations
Addresses a high-priority policy issue	Yes	The capacity of MHSUH providers to address emerging population needs is a high-priority national policy issue
Clear meeting objectives	Yes	Policy dialogue objectives were determined in advance and circulated with the invitation and the pre-meeting information package. Objectives were reiterated at the beginning of the policy dialogue verbally and on a shared-screen slide
Pre-circulated information package and evidence summaries	Yes	Participants were provided with an agenda (see Table 1), slide deck containing synthesized literature review and preliminary study findings, and consent form in advance Materials were available in both French and English, Canada’s two official languages
Environment conducive to deliberations	Yes	Policy dialogue facilitated over Zoom using web-based GDSS technology, shared screens, small breakout rooms facilitated by members of the research team, and an external overarching professional facilitator Three-hour meeting scheduled during business hours across five Canadian time zones One breakout room facilitated in French
Clear rules of engagement	Yes	Chatham House Rule followed Experienced facilitator hired to conduct policy dialogue and train research team in breakout room facilitation and GDSS software use
Recording of discussions	Yes	Main session and breakout rooms in Zoom were recorded Recording prompt on Zoom required participants to provide consent to record to stay in the meeting Written consent forms were provided to all participants in advance of the meeting, and the link provided in the Zoom chat box at the start of the meeting
Representation of various stakeholder perspectives (including researchers and knowledge user partners), including those who may be affected by decisions related to the issue	Yes	Participants were purposively selected to represent government, policy and practitioner stakeholders (see Table 3) Stakeholders included a range of MHSUH providers Participants were assigned to small-group breakout rooms to maximize variation of perspectives The research team (including knowledge user partners/advisors) played a key role of discussion group facilitators
Synthesis of high-quality research evidence	Yes	Synthesis of research findings from literature review, pan-Canadian MHSUH provider survey and key stakeholder interviews were provided in advance of the policy dialogue and presented at the beginning of the dialogue
Opportunity for discussion	Yes	Facilitated small-group breakout rooms included 5–7 participants Combined, two breakout sessions included over 1 hour for discussion
No emphasis on reaching consensus	No	One of the objectives was to assess and foster ‘near’ consensus around the priority policy implications (*Focus 1*) and next steps (*Focus 2*) of the research findings Using an adapted nominal group technique, each group’s top three ideas (based on the small-group discussion) for both focus questions were collated and synthesized into a long list by the expert facilitator, then ranked by individual participants in order by priority With less time and space for generative discussion in a virtual format (versus in-person), this consensus-building exercise allowed for more focused and concrete discussion The research team clearly communicated that these priorities would help direct next steps, but no commitment to specific actions was expected from participants
Skilled facilitation	Yes	External expert facilitator (not a stakeholder or part of the research team) hired to facilitate the main session and lead the ranking and voting Breakout rooms were facilitated by research team members familiar with the subject matter and trained in the use of the GDSS software
Outcome evaluation	Limited	The external expert facilitator provided anonymous post-dialogue evaluation forms to each participant with few completing (*n* = 11/46)
Outputs developed and action plan put in place	In progress	Critical commentary “call to action” article prepared for publication (Bartram et al. [17]) Multiple conference presentations and keynotes conducted Infographic developed of research findings including insights from policy dialogue Webinar conducted as part of the Canadian Health Workforce Network’s annual webinar series (November 2021) Policy dialogue reflection and follow-up with expert advisory group completed (November 2021)

**Table 3:** Profile of stakeholder participants attending the policy dialogue

Organizational sector	Number of participants
Mental health/ substance use he alth organizations	9
Regulators or professional associations	8
Mental health or substance use health service providers	8
Government	8
Other health organizations	6
Academic/research	3
Lived experience/lived experience advocacy organizations	2
Industry (e.g. employment insurance)	2
Total	46

### Participants

We invited participants to attend the policy dialogue based on their knowledge of and interest in the MHSUH workforce. Our research study was guided by a pan-Canadian expert advisory committee composed of knowledge users and collaborating organizations; members of this committee were invited to the policy dialogue and asked to identify further possible attendees. During our qualitative stakeholder interviews, we told participants about the policy dialogue and asked whether they had suggestions of others we should invite. We directed our invitations to senior leaders in government departments and organizations, asking them to suggest delegates if they could not attend.

We prioritized inviting a diversity of stakeholder perspectives as much as was feasible, including across organizational sector, occupation, lived experience, geographic region of Canada and ethno-racial and Indigenous identity. A total of 46 stakeholders representing a variety of sectors from across Canada attended the virtual policy dialogue (see Table 3 for a profile of stakeholder attendees). The policy dialogue followed the Chatham House Rule; thus, we are not reporting any identifying information beyond the organizational sector of each participant.

### Ethics

This research study was approved by the research ethics boards at the University of Ottawa and Athabasca University. Written online consent forms were provided to all participants in advance of the policy dialogue and the link was provided in the Zoom chat at the beginning of the meeting for anyone who had not yet completed the form. A recording prompt on Zoom required participants to consent to the recording to remain in the online meeting room.

### Data collection and analysis

Data generated from policy dialogues come from purposeful conversations where contributors collectively create new understandings by complementing research evidence with their own experiences and knowledge on a particular issue [22]. The data collected from the virtual policy dialogue included the full transcripts from each small-group discussion and the main sessions, reflective field notes from the research team members who facilitated the breakout rooms, and the final report from the external facilitator. This final report captured all the ideas generated in the small groups and the synthesized and prioritized ideas from the main sessions. Audio recordings from the main session and breakout rooms were transcribed verbatim and coded using NVivo.

Studies using policy or deliberative dialogues are unique in that the interpretation of research data by the dialogue participants is, in and of itself, a source of primary data [22]. We developed an initial (*deductive*) coding framework based on the preliminary results of our main research study (see Additional file 1). As Plamandon et al.’s [22] integrated framework for analysing data from deliberative dialogues suggests, we also used the key messages identified as policy dialogue participants engaged with our research as further (*inductive*) codes for categorizing emerging data from the policy dialogue (see Additional file 2). Coded data were thematically analysed to identify key concepts raised in the small-group breakout rooms and in the plenary sessions. Coding and thematic analyses were regularly discussed and shared among the research team for feedback and consensus.

## Findings

In both the pre-circulated research brief and the research team’s presentation at the beginning of the policy dialogue, we presented a synthesis of evidence from our year-long mixed-methods research study that included an extensive literature review, a pan-Canadian MHSUH provider survey and key stakeholder interviews. The main findings of this study are presented elsewhere [16, 17, 29]. A summary of the key research findings discussed in the facilitated virtual policy dialogue is presented in Textbox 1.

Textbox 1: Key research findings discussed in facilitated virtual policy dialogue

**Table:** 

We conducted a literature synthesis, a pan-Canadian survey of 2177 individuals providing MHSUH services, and 18 semi-structured key informant interviews to gain a deeper understanding of the pandemic’s impact on the MHSUH workforce The literature synthesis included 129 published articles and 280 grey literature sources and identified negative impacts of pandemics and disasters on MHSUH workforce capacity or service provision, specific modifications made by MHSUH workforces to better respond to population health needs during crises, and the impact that gender, race, ethnicity and other social identities had on MHSUH population needs, service provision and providers during the COVID-19 pandemic [16] Our pan-Canadian survey found an overall decrease in the capacity of the MHSUH workforce during pandemic despite increasing demands, with the impact varying across occupations, genders and funding models [29] Key informant interviewees identified critical challenges in ensuring MHSUH workforce capacity to respond to increasing demand: adapting to virtual service delivery, providing adequate infrastructure and logistics, recognizing hidden MHSUH occupations, reducing the divide between public and private funding for MHSUH services, preventing provider burnout and addressing workforce data gaps and silos [29]

### Policy dialogue: implications of research findings

The ranking exercises resulted in 38 potential policy implications of the research findings (*Focus 1* of the policy dialogue). Through discussion led by the external facilitator, this list was condensed by collating similar items together and offering participants a chance to respond and discuss individual items. Participants then voted on their top five choices individually and anonymously. The ten top-ranked policy implications are presented in Table 4.

**Table 4:** Ranked policy implications of our research findings (Focus 1)

Rank	Item	Votes
1^a^	Develop a more diverse and culturally competent workforce	31
1^a^	Create environments that prevent MHSUH workforce burnout	31
2	Collect comprehensive MHSUH workforce data, stratified by race, ethnicity, gender, etc.	26
3	Invest in training, recruitment and regulation	25
4^a^	Achieve funding parity between MHSUH and physical health services	22
4^a^	Communicate the need to strengthen MHSUH workforce capacity in response to the pandemic	22
5	Optimize the mix of virtual and in-person delivery to broaden reach	21
6	Create better policy on interface between public and private sectors	16
7^a^	Remove barriers to inter-provincial mobility	15
7^a^	Value contributions of different roles with the MHSUH workforce, including peer support	15

^a^Tie in ranking with another item

### Policy dialogue: action items

Following the discussion and ranking of the policy implications of our research findings, the same process was followed to generate action items based on the identified policy implications, resulting in 42 potential action items. The top three priority action items stemming from each small-group discussion were collated and discussed in the plenary session by the external facilitator and voted on by participants. The 10 top-ranked action items are presented in Table 5.

**Table 5:** Ranked action items based on policy implications (Focus 2)

Rank	Item	Votes
1	Provide full public funding for MHSUH care, including preventative care and care that addresses inequities	27
2	Collect standardized MHSUH workforce data, including demographic data	26
3	Develop competencies and tools for culturally appropriate services	24
4	Generate better MHSUH workforce data, including unregulated providers, with a focus on sex/gender, racial and other equity demographics	21
5	Manage burnout through support, remuneration and integrated care models	20
6	Increase supply through training, remuneration and recruiting for diversity	16
7	Remove regulatory barriers to improve access to quality MHSUH services	14
8^a^	Increase support for community-led interventions	12
8^a^	Adopt promising practices	12
9	Adopt psychological health and safety standards for the MHSUH workforce	11

^a^Tie in ranking with another item

### Final actionable policy recommendations

The thematically analysed transcripts from the small breakout rooms and large group discussions informed our contextualized understanding of the prioritization of the policy implications and action items. We identified five overarching themes from the policy dialogue, with *equity* as a cross-cutting theme that permeated the discussion and all five main themes. Using the prioritized lists created from both *Focus 1* (policy implications of the research findings) and *Focus 2* (action items and next steps), as well as the themes identified in the transcripts, we created a synthesized set of actionable policy recommendations that arise from our research findings (see Table 6).

**Table 6:** Themes and actionable policy recommendations arising from the facilitated virtual policy dialogue

Theme	Actionable policy recommendation	Key points and equity considerations arising from small-group discussions
Funding	Increase public investment in the MHSUH workforce and promote sectoral coordination, particularly between the public and private sectors	MHSUH services in Canada are severely underfunded, creating inequities that significantly impact marginalized and vulnerable groups: Canada needs to achieve funding parity between MHSUH services and physical health services to address these equity issues Preventative MHSUH care should be prioritized and fully funded Public investment is required to address the disparity in remuneration between MHSUH providers (e.g. between providers in public/private sectors and peer support workers) Public investment in the training and recruitment pipelines would help address the increasing MHSUH needs of the population, similar to what some Canadian jurisdictions have done for personal support workers Explicit policy addressing the relationship between the public and private sectors to create more seamless care, provide better integration, optimize synergies and avoid inefficiencies that result from lack of coordination
Regulation and recognition	Standardize regulation across the country to promote equitable access to services and remove barriers to practice, particularly around inter-jurisdictional practice and virtual care	Regulatory barriers hindering inter-provincial virtual care and mobility of regulated practitioners need to be addressed; this would allow the delivery modality (virtual or in-person) to be optimized, recognizing that each modality has potential equitable access implications, and enable the workforce to be where it is needed with more efficient deployment Pandemic has highlighted the importance of regulation in contributing to equitable access to services since much public and private funding is limited to regulated providers Focus on promoting collaboration between providers with complementary scopes of practice to provide patients with comprehensive MHSUH services
Burnout and well-being	Create enabling environments for MHSUH provider well-being and retention to address burnout	Recruitment strategies to increase supply of providers should include a focus on enhancing the diversity of the MHSUH workforce Learn from experiences of other groups of providers who have developed or identified promising practices (e.g. law firms that are identifying metrics contributing to employee burnout) Uniform adoption of standards and positive practices to improve psychological health and safety of MHSUH providers in both public and private sectors, including consideration of unique harms that may occur when providing virtual services The existing peer support community of MHSUH providers with lived MHSUH experience is hidden due to stigma, creating lack of access to care and burnout: MHSUH practitioners should be able to speak freely about their own MHSUH concerns and seek the support of peers without fear of being stigmatized
Workforce data	Develop comprehensive and standardized datasets describing the MHSUH workforce for better workforce planning	We need a clear definition of what is classified as the MHSUH workforce because job titles and descriptions vary across the country Data should be collected in a standardized way across the country to allow for comparison across jurisdictions and provider groups and contribute to systematic workforce planning Data sharing needs to be established so that data does not stay in silos Data collection challenges are particularly prevalent for unregulated MHSUH providers Better data is required to understand who is in the MHSUH workforce to allow Canada to stratify its MHSUH workforce by different identifiers (e.g. race, ethnicity and gender): we cannot manage what we do not measure, and we currently do not measure any of these diversity or equity demographics of the workforce
Cultural competence	Equip MHSUH workforce to provide culturally appropriate and person-centred care	The MHSUH workforce needs to develop the knowledge, skills and competencies to address MHSUH across the continuum of care and services—from promotion and prevention to treatment of serious and concurrent illness Measuring equity and diversity demographics of the MHSUH workforce will contribute to building the capacity of the workforce to provide culturally appropriate and person-centred care More research is needed to identify the barriers to providing culturally appropriate services and identify the core competencies required to provide services for a range of equity-seeking groups, recognizing that different skills are needed to work with different groups Toolkits should be developed to assess MHSUH service teams to understand whether the range of competencies are represented and build on skills already present Interventions to increase cultural competence need to include a diversity of voices and be community-led

## Discussion

Our facilitated virtual policy dialogue allowed our research team to acquire broad input from a diverse group of participating stakeholders and facilitated critical consideration of our research findings. Throughout the small-group breakout room discussions, participants were highly engaged and offered thoughtful insight into the policy implications of our research and key action items, generating rich qualitative data to deepen our understanding of these considerations. The strong agreement by stakeholders on the prioritization exercises suggest that these policy recommendations are critical in addressing the capacity concerns we identified in our research on the MHSUH workforce. As the pandemic continues to challenge the health system and the MHSUH workforce, bridging the evidence–policy divide is critically important.

Tailoring the policy dialogue to the virtual format—while necessitated due to COVID-19 travel restrictions and social distancing requirements—offered some key advantages. These included the ease of brainstorming through shared screen functions, preassigned breakout rooms, automatic transcription and the ability to share links for consent forms and websites in the chat. Recordings were easily done on the Zoom platform, replacing the need for note-takers in each breakout session, and all participants needed to consent to the recording to stay in the meeting. The virtual format was also less resource-intensive (time and money) than an in-person event. This allowed for a variety of voices to be more readily mobilized since stakeholders across Canada could attend more easily, overcoming a criticism of in-person events that can be resource intensive and exclusionary [26]. By making these knowledge mobilization strategies less resource intensive, easier to plan and facilitate, and potentially more inclusive, facilitated virtual policy dialogues may be a valuable mechanism to allow more routine use of evidence to inform policy decision-making. Researchers and policy-makers should consider the broader systems-level advantages of holding these types of dialogues more often and easily via virtual formats.

Other technological advantages of the virtual format included using the web-based GDSS software for brainstorming and ranking ideas and recording the group discussions. Using the GDSS technology allowed for more structured discussion and made it easy to bring ideas together from the various breakout rooms for plenary discussion and prioritization in a virtual environment. Researchers planning virtual policy dialogues should consider factoring in the costs associated with this or similar technology, as well as the costs of engaging an expert external facilitator. The Zoom video platform also allowed for recording and full transcription of each small-group discussion session. We were then able to analyse the rich qualitative data from these transcripts, making for a deeper analysis than would be possible if we only had access to written notes from these groups.

We included as an objective for our policy dialogue to assess and foster consensus regarding the key policy implications and priority action items arising from our research study. We did this through our facilitated prioritization (ranking and voting) exercises, incorporating an adapted nominal group technique as a consensus identification strategy. Each small group’s top three ideas (based on the breakout room discussions) for both focus questions were collated into a long list by the expert facilitator, then ranked in order by priority individually. Generally, research on policy dialogues suggests that participants value not emphasizing the need for consensus since it is unlikely policy-makers or stakeholders would commit themselves to a particular solution without further input and processes [19]. However, through the prioritization exercises, we were trying to move towards consensus on foundational steps required for evidence-informed policy, an outcome possible from knowledge mobilization policy dialogues [20], rather than seeking commitment from stakeholder attendees to specific actions. Building consensus through dialogue can be particularly valuable in the MHSUH context given that there is a broad spectrum of stakeholders and—in Canadian MHSUH policy—accountability is spread across many sectors and levels of government [21]. In future policy dialogues, we suggest it may be valuable to phrase this objective as *understanding policy implications from the research and developing a consensus-driven list of actionable solutions* to address this variation from conventional policy dialogue guidance. Priority-setting approaches may be particularly valuable in a virtual setting, where time and the power of in-person connection are constrained. At the same time, participants may feel that priority-setting is rushed or forced in a virtual dialogue.

Following the policy dialogue, we held a meeting to develop a call-to-action paper and infographic, building on the priorities and themes identified. We also incorporated these into a webinar conducted as part of the Canadian Health Workforce Network’s annual webinar series. Along with more traditional research deliverables of peer-reviewed journal articles and conference presentations, these created a diverse and integrated knowledge translation strategy for our study. Working with stakeholders in this way also allowed us to grow our network for individuals and organizations from across Canada who have knowledge, experience and interest in the MHSUH workforce and who continue to work with us on our research in this area.

### Limitations

Our virtual policy dialogue had some limitations. Despite the advantages of the virtual format, some participants may have been less comfortable with the Zoom video conferencing technology or may have had challenges with access due to Internet connectivity issues. Given the capacity issues that we identified in our study, we also knew that many stakeholders would have pandemic-induced fatigue or burnout, including around attending virtual events. We kept the dialogue to 3 hours with this in mind and offered ways for stakeholders to stay engaged with the research following the policy dialogue. Nevertheless, a full-day event may have generated further discussion and ideas.

We obtained feedback from participants at the end of the virtual policy dialogue via a quick online survey. However, only 11 participants completed this, making the data of limited value. It may have been helpful to send a follow-up survey to assess how participants viewed the virtual policy dialogue process.

Finally, although we made a serious effort to invite a diverse range of stakeholders, we were not able to engage with all potentially impacted MHSUH groups. The resulting interpretations of our research in terms of policy implications and actionable solutions only reflect the views of those who were in the virtual room and are thus constrained by a potential lack of diversity in providers, perspectives and lived experiences.

### Future directions

Adapting conventional in-person policy dialogues to virtual formats may offer researchers a way to engage a greater diversity of voices with study findings and more routinely use evidence to inform policy decision-making. Future studies using facilitated virtual policy dialogues as a knowledge mobilization strategy could incorporate ways to evaluate the process (focusing on the modifications required for virtual formats and how best to promote inclusion and participation) and outcomes (focusing on subsequent engagement by stakeholders with the generated policy recommendations). Our inclusion of an adapted nominal group technique and interactive GDSS technology should be evaluated as an evolution of policy dialogues that potentially allows for more equitable participation by stakeholders and may help foster consensus on next steps.

## Conclusions

Facilitated virtual policy dialogues provide rich qualitative insights that can guide a community-informed knowledge mobilization strategy and promote evidence-informed policy. Our facilitated virtual policy dialogue was a key knowledge mobilization strategy in our mixed-methods study on the capacity of the Canadian MHSUH workforce to respond to the COVID-19 pandemic. As the final phase of our research, this facilitated virtual policy dialogue allowed us to engage a diverse group of MHSUH workforce stakeholders in a meaningful action-oriented way, provided an avenue to get feedback on our research findings, and generated prioritized action items that incorporated the knowledge and experience of these MHSUH workforce stakeholders.

## Supplementary Information

**Additional file 1**: Initial (*deductive*) coding scheme for the policy dialogue based on main research study.

**Additional file 2**: Adapted (*inductive*) coding scheme for the policy dialogue.

## Data Availability

Portions of redacted transcripts may be made available from the corresponding author on reasonable request.
