# Supplementary material for: Enhancing the capacity of the mental health and substance use health workforce to meet population needs: insights from a facilitated virtual policy dialogue
Source: Health Res Policy Syst. 2022 May 7;20:51. doi: 10.1186/s12961-022-00857-8 (PMC9077339; doi:10.1186/s12961-022-00857-8)
Supplement: Supplementary file 1 — Additional file 1: Initial (deductive) coding scheme for the policy dialogue based on main research study. [file 12961_2022_857_MOESM1_ESM.pdf]

ADDITIONAL FILE 1: Initial (*deductive*) Coding Scheme for the Policy Dialogue based on Main Research Study

1. PANDEMIC IMPACT

a. Impact on changing population needs
b. Specific to subsector
c. Impact on capacity
    i. increased capacity,
    ii. enablers to increasing capacity (from survey)
        1. virtual care
        2. rapid referrals
        3. volunteering time
        4. reduced admin barriers
        5. reduced regulatory barriers
        6. increased funding
        7. change in workload allocation
        8. more individuals seeking services
        9. practicing to optimal scope
        10. additional/refresher training
        11. additional coping resources and supports
    iii. unchanged capacity,
    iv. decreased capacity
    v. barriers that have reduced capacity (from survey)
        1. lockdown
        2. no access to virtual care infrastructure
        3. clients lack access or are uncomfortable with virtual care, less people seeking services
        4. COVID-19 protocols slow down service
        5. need training in virtual care
        6. change in workload
        7. limited access to PPE
        8. redeployment
        9. personal responsibilities
        10. own MHSU concerns:
            a. burnout or stress
            b. turnover & early retirement
            c. leaving for private sector
d. Change from first pandemic wave to subsequent waves
    i. initial impact
    ii. ongoing impact

2. SOURCE OF DATA ON MHSU WORKFORCE NEEDS & CAPACITY DURING THE PANDEMIC

a. Focused population level data
    i. Demographic characteristics (including equity data) on population
b. Mental health and substance use workforce capacity
    i. Data sources
    ii. Human resources data
    iii. Anecdotal data
    iv. Qualitative data from team meetings
    v. Surveys
    vi. Registry
c. Data silos
    i. Public/private
    ii. Regulated/unregulated
d. Data gaps
    i. Data and evidence lacking for MHSU worker groups
        1. Mental health (generally)
        2. Substance use (generally)
        3. Specific occupation
    ii. Types of data
        1. Demographic characteristics (including equity data) on workers/workforce
        2. Geographic participation
        3. Activity and participation rates
        4. Scope of practice
e. Data accessibility
f. Data wishes
