# Supplementary material for: Enhancing the capacity of the mental health and substance use health workforce to meet population needs: insights from a facilitated virtual policy dialogue
Source: Health Res Policy Syst. 2022 May 7;20:51. doi: 10.1186/s12961-022-00857-8 (PMC9077339; doi:10.1186/s12961-022-00857-8)
Supplement: Supplementary file 2 — Additional file 2: Adapted (inductive) coding scheme for the policy dialogue. [file 12961_2022_857_MOESM2_ESM.pdf]

ADDITIONAL FILE 2: Adapted (*inductive*) Coding Scheme for the Policy Dialogue

1. **PANDEMIC IMPACT**
    a. Impact on changing population needs
    b. Specific to subsector
    c. Impact on capacity
        i. Increased capacity
        ii. Unchanged capacity
        iii. Decreased capacity
    d. Change from first pandemic wave to subsequent waves
        i. Initial impact
        ii. Ongoing impact

2. **SOURCE OF DATA ON MHSU WORKFORCE NEEDS & CAPACITY DURING THE PANDEMIC**
    a. Focused population level data
    b. Mental health and substance use workforce capacity
    c. Data silos
    d. Data gaps
    e. Data accessibility
    f. Data wishes

3. **IMPLICATIONS AND RECOMMENDED RESPONSES TO PANDEMIC IMPACTS**
    a. Broader societal implications
    b. Broader system implications
    c. Broader system implications
    d. Data wishes
    e. Specific to data
    f. Specific to equity-diversity-inclusion implications
    g. Specific to profession/occupation group
    h. Specific to sector
    i. Specific to team/organization
